# Observational Cohort Study of TetraGraph^®^ Electromyography Compared to Standard Acceleromyography Monitoring

**DOI:** 10.3390/jcm14176245

**Published:** 2025-09-04

**Authors:** Stass Danielsons, JoEllen Welter, Alexander Dullenkopf

**Affiliations:** Institute of Anesthesia, Spital Thurgau Frauenfeld, 8501 Frauenfeld, Switzerland; stass.danielsons@stgag.ch (S.D.); joellen.welter@stgag.ch (J.W.)

**Keywords:** anesthesia, patient safety, monitoring, neuromuscular blockade

## Abstract

**Background/Objectives**: Current guidelines recommend objective neuromuscular monitoring to ensure patient safety during neuromuscular blockade. Acceleromyography using train-of-four (TOF) stimulation is most commonly used to assess neuromuscular function. This study compares a new electromyography-based monitor with an established acceleromyography device for neuromuscular monitoring when mounted on a restricted arm. **Methods**: This prospective, controlled observational study enrolled patients undergoing surgery with general anesthesia requiring neuromuscular blockade. Two neuromuscular monitoring systems were used simultaneously: the standard acceleromyography device (Philips IntelliVue MX550) and the electromyography-based TetraGraph^®^ monitor on the opposite arm. Atracurium was administered as the neuromuscular blocking agent. The TetraGraph^®^ arm was restricted during surgery. The primary outcome was the time for the TOF ratio to return to ≥90%. Secondary endpoints included the time to reach a TOF count of 0 during induction. Data were analyzed using Bland–Altman plots and a paired *t*-test. **Results**: Mean time to recovery to TOF ratio ≥ 90% was 67 min (±21.4) for IntelliVue MX 550 and 75.8 min (±22.3) for TetraGraph^®^ (*p* = 0.0001; mean bias 8.9 min, 95% confidence intervals (CIs) 5.99–11.8). The mean time to reach a TOF count of 0 was 180.6 s (±7.8) for IntelliVue and 200 s (±8.2) for TetraGraph^®^ (*p* = 0.0217; mean bias 19 s, 95% CI 2.96–35.8). **Conclusions**: TetraGraph^®^ consistently recorded the endpoints later than IntelliVue, reflecting slower onset and recovery times. However, substantial intra-individual variability was observed with both devices during recovery from neuromuscular block. The observed differences may have clinical implications, such as when assessing readiness for extubation.

## 1. Introduction

The administration of neuromuscular blocking agents (NMBAs) is a standard anesthetic practice aimed at facilitating tracheal intubation and improving operative conditions. To minimize the risks of residual neuromuscular blockade and associated complications, clinical guidelines strongly advise the use of quantitative neuromuscular monitoring techniques [1,2].

While technologies such as mechanomyography (MMG) and electromyography (EMG) are regarded as reference methods for assessing neuromuscular transmission, their technical demands have limited their routine application in the operating room [3,4]. In contrast, acceleromyography (AMG), relying on the acceleration of a certain muscle as a surrogate for force, has gained widespread acceptance in the operating room due to its simpler setup and real-time feedback capabilities. As such, AMG is currently regarded as the standard tool for neuromuscular monitoring in routine anesthesia practice [5].

In clinical neuromuscular monitoring, stimulation is typically applied to the ulnar nerve at the wrist [3]. The resulting thumb movement, in the form of acceleration, is measured as an indicator of muscle response. Among the various stimulation protocols available, the train-of-four (TOF) technique is the most widely utilized. This method involves delivering four sequential electrical pulses and assessing the muscle responses, specifically by comparing the response to the fourth impulse to that of the first. The resulting TOF ratio, expressed as a percentage, ranges from 100% (indicating no effect of neuromuscular blockade) to 0% (indicating absence of a detectable fourth response). When the fourth twitch is no longer detectable, the number of remaining twitches is recorded as the TOF count. Complete suppression of all four twitches corresponds to a TOF count of 0, typically targeted during induction for optimal intubation conditions. However, complete blockade is not always required intraoperatively; a TOF count of 2 is often considered sufficient for most surgical procedures [6]. Neuromuscular recovery was generally defined by a TOF ratio equal to or exceeding 90% with newer guidelines recommending TOF ratio 100% when non-normalized AMG monitoring is used [1,2,7,8]. As AMG typically assesses acceleration and therefore movement of the thumb, the method is dependent on the ability of the thumb to move in an unrestricted manner. Conditions are unfavorable when the movement of the thumb is restricted or the extremity cannot be observed.

The TetraGraph^®^ monitor (Senzime, Uppsala, Sweden) is a recently introduced device that utilizes EMG to assess neuromuscular function [9,10,11,12]. While its external design resembles traditional AMG monitors, TetraGraph^®^ measures electrical signals from the *adductor pollicis* muscle in response to ulnar nerve stimulation, rather than detecting physical thumb movement. According to the manufacturer, the system is designed for ease of setup and use, and does not require precise positioning of the forearm, potentially improving practicality in the clinical setting.

Despite its introduction into routine practice, there is limited clinical data comparing TetraGraph^®^ with established neuromuscular monitoring systems. This study was conducted to evaluate the performance of the TetraGraph^®^ EMG monitor in comparison to a widely used AMG-based device (IntelliVue MX550; Philips, Horgen, Switzerland) with TetraGraph^®^ mounted on a restricted arm during surgery.

The primary outcome was the time required for recovery to a TOF ratio of at least 90% following neuromuscular blockade. Secondary outcomes included the time to achieve complete suppression of the TOF ratio (0%) during induction, and the time to return to a TOF count of 2 during recovery. We hypothesized that the measurements obtained from both devices would be comparable across all assessed outcomes.

## 2. Materials and Methods

### 2.1. Study Design

This prospective, controlled observational study was conducted in accordance with the ethical standards of the institutional and/or national research committee and with the 1964 Helsinki Declaration and its later amendments or comparable ethical standards. The study was performed with the approval of the Ethics Committee of Eastern Switzerland (BASEC No. 2024-D0055) and was registered in the German Clinical Trials Register (DRKS00034376; 18 June 2024). Prior to participation, all subjects received detailed information regarding the study procedures and provided written informed consent. The setting of the study was a cantonal hospital in northeastern Switzerland.

Adult patients scheduled for elective surgical procedures under general anesthesia from June 2024 through April 2025 at our institution were prospectively screened for eligibility. Inclusion criteria required the planned use of non-depolarizing NMBAs in accordance with institutional anesthesia protocols. In addition, only patients undergoing operations that necessitated restriction of one upper limb—rendering it inaccessible to the anesthesia team during the procedure—were eligible for inclusion. Examples include robotic-assisted surgeries, laparoscopic cholecystectomies, or hernia repairs.

Exclusion criteria included emergency surgeries, the need for rapid sequence induction, or the presence of any contraindication to the use of atracurium. Individuals with known neuromuscular disorders, morbidly obese persons or those who were pregnant were also excluded. Patients already enrolled in this trial or concurrently participating in any other research study were not eligible for inclusion.

Neuromuscular transmission was monitored simultaneously using two devices—IntelliVue and TetraGraph^®^—placed on the same patient. The primary outcome was the duration from administration of a neuromuscular blocking agent to full recovery, defined as the achievement of a train-of-four ratio of ≥90%. Secondary outcomes included the time to complete suppression of twitch response (TOF ratio of 0%) during induction, and the time required for return of a TOF count of 2 during recovery. In all patients, the skin was properly prepared before stimulation electrodes were applied according to the manufacturer’s instructions.

The TetraGraph^®^ device was attached to the restricted arm. During anesthesia induction, however, both arms remained accessible and were appropriately positioned to allow accurate AMG recordings. According to institutional guidelines, the unrestricted arm is typically used for infusion; therefore, blood pressure was measured on the restricted arm. All clinical decisions during anesthesia were based exclusively on data from the IntelliVue system, which serves as the standard monitoring tool at the institution.

For all study patients, the IntelliVue system served as the reference or control device. For calibration, the ulnar nerve was stimulated to identify the supramaximal threshold with the thumb secured and constant preload applied using a dedicated positioning device (IntelliVue NMT Hand-Adapter; Philips, Horgen, Switzerland) to ensure consistent measurement conditions. The IntelliVue monitor recorded TOF responses at a minimum interval of 12 s. The TetraGraph^®^ monitor was evaluated as the experimental or investigative device. It was applied to the opposite arm, where stimulation of the ulnar nerve was performed using the manufacturer’s default setting of 50 mA for manual operation. The device captured neuromuscular responses at its minimum allowable interval of 20 s between TOF stimulations.

### 2.2. Anesthetic Management

Prior to surgery, patients received oral premedication with 7.5 mg of midazolam approximately 30 min before anesthetic induction. Upon arrival in the operating room, standard monitoring was initiated, including electrocardiography (ECG), non-invasive blood pressure (NIBP), and pulse oximetry (SpO_2_). Depth of anesthesia was monitored using bispectral index (BIS) analysis via processed EEG analysis (Philips, Zurich, Switzerland). An intravenous catheter was placed for administration of fluids and medications.

The TetraGraph^®^ neuromuscular monitoring device was applied to the arm expected to be restricted during surgery, typically positioned against the patient’s body after induction. The IntelliVue system was attached to the opposite limb, which in most cases also served as the infusion site. Following placement of monitors, patients underwent pre-oxygenation and received intravenous fentanyl at a dose of 1.5 mcg/kg. Anesthesia was then induced using target-controlled infusion (TCI) of propofol, with an effect-site concentration (Ce) set to 6 mcg/mL. The protocol allowed for the use of remifentanil via TCI at a Ce of 2 mcg/mL, either as an adjunct or alternative, depending on clinical requirements.

After loss of consciousness, both neuromuscular monitors were activated simultaneously. IntelliVue calibration was performed at this time, and baseline TOF measurements were recorded from both devices prior to administration of neuromuscular blockade. Stable conditions were defined as three consecutive, consistent TOF measurements from both devices. Atracurium, the only neuromuscular blocker used, was refrigerated and administered according to manufacturer labeling and institutional pharmacy procedures. This non-depolarizing NMBA was given intravenously at a dose of 0.5 mg/kg. Anesthesia was maintained with a propofol-based TCI regimen and supplemented with sevoflurane and/or additional opioid dosing as needed. Further doses of atracurium were administered during the procedure at the discretion of the anesthesiologist. There was no pharmacological reversal of atracurium during the study.

### 2.3. Data Collection

Baseline demographic characteristics and anesthetic management details were collected from the electronic medical records of each patient. The specific side on which each neuromuscular monitor (TetraGraph^®^ and IntelliVue) was applied was also documented. During induction of anesthesia, TOF measurements from both devices were captured at 30 s intervals, starting after calibration of IntelliVue and commencing until a TOF ratio of 0% was confirmed by three consecutive readings on both monitors. If a device was mid-measurement at the time of a scheduled reading, the value was recorded immediately after the measurement completed.

Following this stabilization phase, TOF values were documented at 5 min intervals until both devices registered a TOF ratio of ≥90%, indicating recovery of neuromuscular function. Additionally, the time from the final dose of atracurium to the reappearance of a TOF count of 2 or more, as well as the time to achieving a TOF ratio of at least 90%, was recorded separately for each monitoring system.

Any technical malfunctions or irregularities encountered with either device during the study were noted. At the conclusion of anesthesia, patients were evaluated for potential skin irritation or adverse dermatologic reactions related to the placement of the neuromuscular monitoring electrodes.

### 2.4. Statistical Analysis

Differences in the timing of neuromuscular blockade onset and recovery between the IntelliVue and TetraGraph monitors were analyzed using Bland–Altman plots, which presented the mean bias and 95% limits of agreement. To assess whether these differences significantly deviated from zero, a one-sample *t*-test was applied. The assumption of normal distribution for the paired differences was evaluated visually using Q–Q plots.

To compare the incidence of technical issues between the two devices, Fisher’s exact test was used due to the categorical nature of the data. Additionally, the reliability of the TetraGraph system was assessed by calculating its predictive accuracy, with IntelliVue serving as the reference standard. Specifically, we determined the number of instances in which TetraGraph^®^ indicated full neuromuscular recovery (TOF ratio ≥ 90%) while IntelliVue still showed residual blockade.

Enrollment proceeded consecutively without a pre-specified sample size. Therefore, we conducted a post hoc sensitivity analysis of the minimum detectable difference for the paired primary endpoint, deriving the standard deviation of the paired differences from the 95% confidence interval (10.22 min) and estimating power at a two-sided significance level of 0.05 with a sample size of 50. Data were analyzed in Stata, version 15.0 (StataCorp, College Station, TX, USA).

## 3. Results

Baseline demographic data of the 50 patients enrolled in the study are presented in Table 1.

The TetraGraph^®^ device was mounted on the right arm in 62% of cases. Anesthesia was induced in all patients using target-controlled infusion (TCI) of propofol and fentanyl. Supplemental remifentanil was administered in 49 patients (98%). During anesthesia induction, a median dose of 40 mg atracurium (interquartile range (IQR) 35–50) was given intravenously, corresponding to a mean of 0.56 mg/kg (±0.1). Tracheal intubation was successful on the first or second attempt in all patients. Throughout anesthesia, sevoflurane was supplemented in 7 cases.

**Figure 1:**
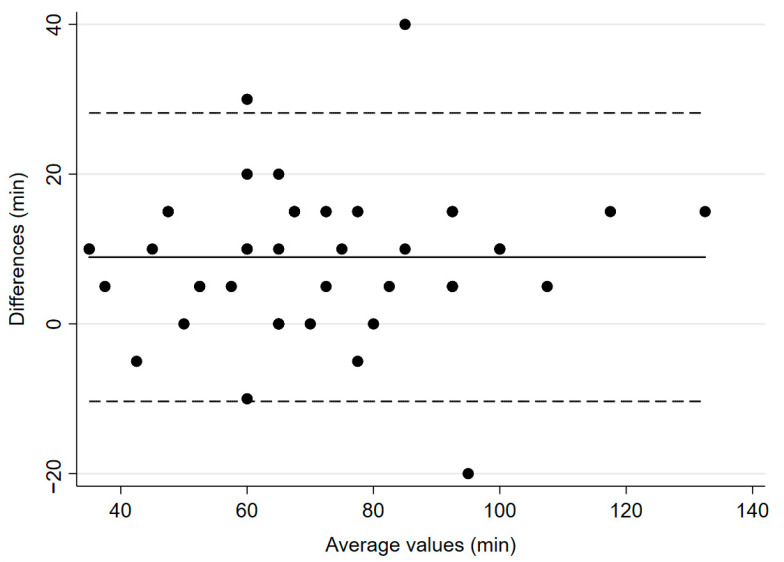
Bland–Altman plot for time to recovery to TOF ratio ≥ 90%. The solid line illustrates the mean difference and the dashed lines indicate average difference ± 1.96 standard deviation of the difference.

The study’s primary endpoint was the mean time to recovery of TOF ratio ≥ 90% from administration of the last dose of atracurium. This value was 67 min (±21.4) for the reference device, IntelliVue, and 75.8 min (±22.3) for the experimental device, TetraGraph^®^ (*p* = 0.0001, Figure 1). The mean bias was 8.9 min and 95% confidence interval (CI) was 5.99–11.8 min.

**Figure 2:**
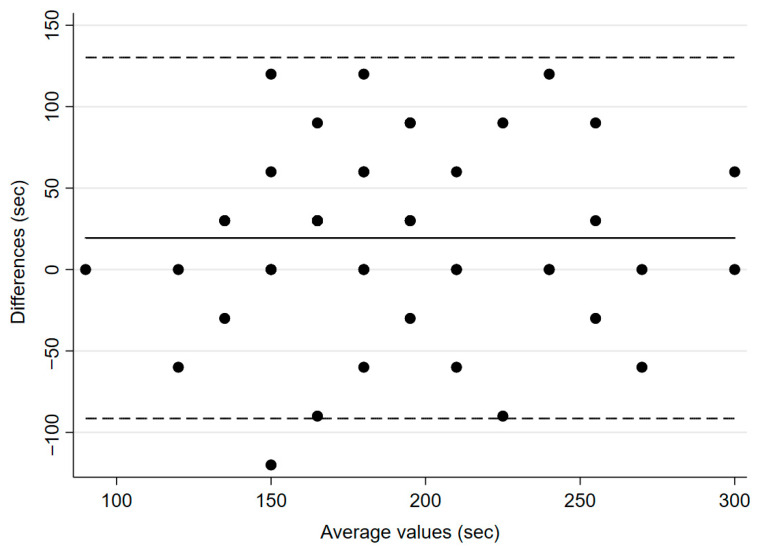
Bland–Altman plot for time to neuromuscular block (TOF ratio 0%). The solid line illustrates the mean difference and the dashed lines indicate average difference ± 1.96 standard deviation of the difference.

During induction, the TOF ratio before administering atracurium, but after calibration of IntelliVue, was 116.0% ± 10.3 for IntelliVue, and 102.4% ± 6.2 for TetraGraph^®^ (*p* < 0.0001). The mean time from administration of the NMBA to a TOF-ratio of 0% was 180.6 s (±7.8) for IntelliVue and 200 s (±8.2) for TetraGraph^®^ (*p* = 0.0217, Figure 2), with a mean bias of 19 s and 95% CI of 2.96–35.8.

**Figure 3:**
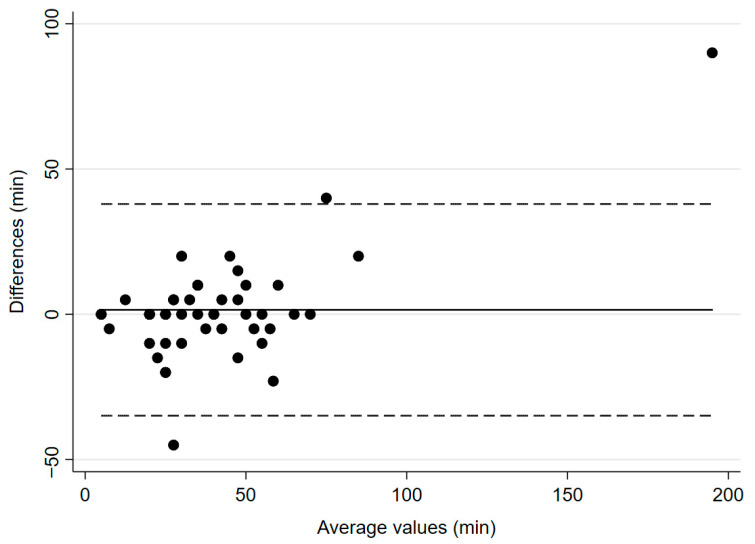
Bland–Altman plot for time to recovery to TOF count ≥ 2. The solid line illustrates the mean difference and the dashed lines indicate average difference ± 1.96 standard deviation of the difference.

The mean time to recovery of TOF count ≥ 2 was 39.9 ± 23.6 min for IntelliVue and 41 ± 36.4 min for TetraGraph^®^ (*p* = 0.8794, Figure 3), with a mean bias of 1.5 min and 95% CI of −3.9–6.99 min. Regarding predictive accuracy, among the 46 patients with valid data, TetraGraph^®^ simultaneously identified recovery (TOF ≥ 90%) in 6 cases (13%). In 36 patients (78%), recovery was identified later by TetraGraph^®^, with the largest observed difference being 40 min.

Technical problems occurred in 10% of cases, including error warnings or failure to obtain measurements (n = 0 with the IntelliVue and n = 5 with TetraGraph^®^; *p* = 0.0563). Failures consisted of device error warnings or an inability to obtain a train-of-four tracing despite stimulation attempts. In three patients, failure occurred during anesthesia induction. Likely contributors include suboptimal electrode–skin contact or high skin impedance. In the remaining two patients, monitoring during anesthesia induction was uneventful with failure occurring during surgery. Additional contributors in these patients might be limb positioning or restricted access under drapes, or cable tension. No evidence of tissue injury or skin irritation was observed at the monitoring sites following device application and measurements.

Lastly, the sensitivity analysis indicated that, with a sample size of 50 and a standard deviation of paired differences of 10.22 min, the minimum detectable mean difference was 4.1 min for 80 percent power and 4.8 min for 90 percent power at a two-sided significance level of 0.05. Accordingly, the study had approximately 93 percent power to detect a 5 min difference.

## 4. Discussion

In this study, we evaluated the performance of two neuromuscular monitoring systems during routine clinical anesthesia. Specifically, we compared (1) the time required for recovery from neuromuscular blockade and (2) the time to onset of blockade during induction. The main finding was that the electromyography-based monitor, TetraGraph^®^, which was placed on a restricted arm following induction, consistently recorded both endpoints later than the acceleromyography-based IntelliVue device, which was applied to the unrestricted arm. Furthermore, the predictive accuracy of the TetraGraph^®^ monitor compared to the standard device was limited.

The IntelliVue neuromuscular monitor system is integrated into the Philips vital signs platform and uses acceleromyography to assess neuromuscular functioning. One advantage of this approach is that it does not require a separate stand-alone device, unlike conventional AMG monitors such as the well-established TOF-Watch^®^ [10]. Nevertheless, effective use of the IntelliVue requires that the patient’s thumb be free to move, which may not be feasible in all surgical contexts. Conversely, the electromyography-based TetraGraph^®^ monitor may offer greater flexibility, as it can be applied to an arm that is restricted or inaccessible during surgery. This feature could become increasingly relevant given the increased use of laparoscopic and robotic procedures, which often involve limited access to the upper extremities. Despite this potential advantage, TetraGraph^®^ failed to acquire neuromuscular measurements in 10% of cases due to technical issues. To reduce failures, mitigation strategies may include optimizing skin–electrode contact (prep/replace), ensuring adequate stimulus or auto-current mode, avoiding cable strain, verifying ulnar-nerve placement with thumb immobilization, and repeating the measurement.

Neuromuscular blockade, induced by non-depolarizing neuromuscular blocking agents, is a routine component of modern anesthesia. International guidelines strongly recommend quantitative monitoring in patients receiving NMBA, especially during the recovery phase, to reduce the risk of residual blockage. Sufficient recovery for safe tracheal extubation is commonly defined as a TOF ratio of at least 90% [1,2].

Historically, AMG has served as the standard for both clinical and research applications due to its ease of use, despite the superior precision of MMG and EMG. MMG and EMG technologies are often considered impractical in routine settings due to the technical complexity and limitations in available neuromuscular monitors [3,5].

Recent advancements have led to the development of commercially available EMG monitors with integrated, user-friendly electrodes and modalities similar to AMG devices [6]. The performance of these newer EMG monitors is comparable to MMG and are increasingly used in clinical practice [9,10]. This shift is noteworthy, as previous studies have highlighted the limitations of AMG, including reduced reproducibility and lower accuracy compared to MMG and EMG [6,7,12,13,14]. One well-documented issue with AMG monitors is its tendency to produce TOF ratios exceeding 100% in patients prior to NMBA administration—a phenomenon known as “overshoot” or “inverse fade” [13]. This effect casts doubt on the reliability of using TOF ratio ≥ 90% as a definitive indicator of full neuromuscular recovery, especially in patients with baseline TOF ratios > 110%.

The overshoot observed with AMG has been attributed to inconsistent thumb positioning during initial stimulation, which may result in an inadequately elevated fourth twitch response. Although we used a specialized brace to stabilize the thumb and forefinger during monitoring, this did not eliminate the problem. In our study, the mean of baseline TOF ratio recorded by IntelliVue was 116%, suggesting a high likelihood of overestimating recovery in a substantial portion of the patients.

During anesthesia induction, the IntelliVue monitor reached a TOF count of 0 faster than the TetraGraph^®^ device. Based on standard pharmacological references and textbooks, the onset of full neuromuscular blockade following administration of atracurium is normally expected within approximately three minutes. In our study cohort, the mean time was 180.6 s with IntelliVue and 200 s with TetraGraph^®^. While this difference may be of limited clinical significance during routine induction, it may be more consequential in time-sensitive situations such as rapid sequence induction. The faster onset recorded by IntelliVue could also reflect reduced sensitivity to neuromuscular blockade, warranting further investigation.

However, during the recovery phase, the IntelliVue consistently registered both the return to a TOF count of 2 (regarded as sufficient for most surgical procedures) and the definite endpoint for recovery from NMBA, TOF ratio ≥ 90% earlier than TetraGraph^®^. These findings align with prior research suggesting that AMG-based monitors tend to overestimate recovery [6,13,15]. This issue has been recognized in recent European guidelines, which now recommend defining complete train-of-four ratio of 100% when using non-normalized acceleromyography data.

The mean onset of TOF count = 0 was 19 s earlier with IntelliVue (180.6 vs. 200.0 s), which is unlikely to change routine management. By contrast, the mean recovery of TOF ratio ≥ 90% was 8.9 min earlier with IntelliVue (67.0 vs. 75.8 min), which could affect extubation timing. This may have consequences in high-risk patients (e.g., obstructive sleep apnea, obesity, frailty) and in shorter ambulatory cases. The clinical impact is likely small in long operations or in settings that use rapid pharmacologic reversal.

Although these differences may have implications for clinical decision-making, they should be interpreted with caution. Substantial intra-individual variability was observed with both monitoring devices, and in a number of cases. This variation highlights the importance of considering patient-specific factors and device performance limitations when interpreting neuromuscular monitoring data. Notably, despite reaching the endpoints of the study later in general, in 4 of 47 evaluable cases (9%) TetraGraph^®^ indicated a train-of-four ratio ≥ 90% while IntelliVue still showed residual block. This discordance has safety implications. It should prompt conservative interpretation alongside the anesthetic regimen and clinical signs.

Limitations of this study include the small, relatively uniform cohort and the absence of a pre-specified sample size, which restrict generalizability and may underpower some endpoints. The sensitivity analysis is post hoc and cannot replace prospective planning; it only quantifies the smallest differences. For the primary endpoint, power was approximately 93% to detect a 5 min difference (minimum detectable mean difference 4.1 min at 80% and 4.8 min at 90%). Moreover, we used AMG monitoring with the IntelliVue system as the reference method, although mechanomyography and electromyography are widely considered the gold standards in neuromuscular monitoring. Nevertheless, AMG remains the most commonly used modality in clinical practice and, as a component of the widely implemented IntelliVue platform, reflects real-world monitoring conditions. While the previously validated AMG device, TOF-Watch^®^, has served as a reference standard in many studies, it is no longer available in Switzerland.

Additionally, we did not apply the TetraGraph^®^ in the manufacturer’s auto-mode, which automatically adjusts stimulation current and intervals. We chose manual mode to synchronize train-of-four stimulations between devices for a direct comparison; however, this may limit external validity. Auto mode, recommended in clinical practice, adjusts the stimulus current to the skin electrode impedance and may reduce measurement failures. We also performed contralateral arm monitoring and did not assess neuromuscular function by EMG at the first dorsal interosseus muscle, which some consider the preferred EMG site or an alternative gold standard for MMG [14]. The TetraGraph^®^ system; however, is specifically designed for single-site monitoring at the thumb. Opposing-arm placement can introduce physiologic asymmetry (perfusion and temperature), especially when one arm is restrained, potentially confounding device comparisons. We started measurements in both devices before blockade while both arms were accessible, synchronized train-of-four stimulations, and recorded simultaneously. Nonetheless, cross-arm effects cannot be excluded. A randomized crossover with arm switching with assessment of skin temperature and randomized blood pressure site would be preferable with a study aim of isolating device effects.

Furthermore, our findings are specific to atracurium and may not generalize to other neuromuscular blockers with different pharmacokinetic and recovery characteristics. Although the paired design reduces between-patient variability, agent-specific pharmacodynamics could still influence device differences. Volatile anesthetics can potentiate neuromuscular blockade. Since 7 of 50 patients received sevoflurane in addition to propofol, reflecting clinical practice, we cannot determine whether differences between devices were the same under total intravenous versus combined anesthesia. Finally, as with most research on this topic, neuromuscular function was monitored at the adductor pollicis, a peripheral site.

## 5. Conclusions

In conclusion, under routine clinical conditions, measurements from the recently introduced electromyography-based monitor, TetraGraph^®^, were associated with modestly delayed onset and recovery of neuromuscular blockade compared to those from a standard acceleromyography-based device. These discrepancies in measurements may have implications for anesthetic management and therapeutic decisions.

## Figures and Tables

**Table 1:** Patient characteristics. Data are presented as mean (±SD) *, median (IQR) or n.

Characteristic	Unit	Result
Age *	years	59.6 (±17.4)
Gender	female	28
ASA physical status *	score (I–IV)	2.3 (±0.5)
Height *	meters	1.69 (±0.1)
Weight	kg	75 (69–87)
Body mass index (BMI)	kg/m^2^	26.6 (23.7–29.7)
Propofol	mg	963.5 (732.2–1415)
Fentanyl *	mg	0.40 (±0.13)
Remifentanil	g	0.91 (0.61–1.2)
Atracurium	mg	60 (45–80)
Surgical Type:	number	
General surgery		24
Gynecology		18
Urology		4
Orthopedics		3
Plastic surgery		1

ASA = American Society of Anesthesiologists.

## Data Availability

De-identified, patient-level paired measurements used to generate the Bland–Altman plots are available from the corresponding author upon reasonable request.

## Abbreviations

The following abbreviations are used in this manuscript:

AMG	Acceleromyography
BIS	Bispectral index
Ce	Effect-site concentration
CI	Confidence interval
EMG	Electromyography
NMBA	Neuromuscular blocking agent
MMG	Mechanomyography
TCI	Target-controlled infusion
TOF	Train-of-four
